# First- and Third-Trimester Urinary Phthalate Metabolites in the Development of Hypertensive Diseases of Pregnancy

**DOI:** 10.3390/ijerph182010627

**Published:** 2021-10-11

**Authors:** Sabrina M. Bedell, Grace R. Lyden, Sheela Sathyanarayana, Emily S. Barrett, Kelly K. Ferguson, Ashley Santilli, Nicole R. Bush, Shanna H. Swan, Thomas F. McElrath, Ruby H.N. Nguyen

**Affiliations:** 1 Department of Women’s Health, Obstetrics and Gynecology, University of Minnesota, Minneapolis, MN 55454, USA; bedells@ccf.org; 2 Division of Gynecologic Oncology, Women’s Health Institute, Cleveland Clinic Foundation, Cleveland, OH 44106, USA; 3 Division of Biostatistics, School of Public Health, University of Minnesota, Minneapolis, MN 55454, USA; lyden017@umn.edu; 4 Department of Pediatrics, Environmental and Occupational Health Sciences, University of Washington, Seattle, WA 98145, USA; sheela.sathyanarayana@seattlechildrens.org; 5 Seattle Children’s Research Institute, Seattle, WA 98121, USA; 6 Department of Biostatistics and Epidemiology, Rutgers School of Public Health and Environmental and Occupational Health Sciences Institute, Rutgers University, Piscataway, NJ 08854, USA; esb104@eohsi.rutgers.edu; 7 Epidemiology Branch, National Institute of Environmental Health Sciences, Durham, NC 27709, USA; kelly.ferguson2@nih.gov; 8 Department of Neurology, Mayo Clinic, Rochester, MN 55904, USA; santilli.ashley@mayo.edu; 9 Departments of Psychiatry and Behavioral Sciences and of Pediatrics, University of California at San Francisco, San Francisco, CA 94143, USA; Nicole.Bush@ucsf.edu; 10 Department of Preventive Medicine and Public Health, Icahn School of Medicine at Mount Sinai, New York, NY 10029, USA; shannahswan@gmail.com; 11 Department of Epidemiology, Harvard T.H. Chan School of Public Health, Division of Maternal-Fetal Medicine, Brigham and Women’s Hospital, Boston, MA 02115, USA; tmcelrath@bwh.harvard.edu; 12 Division of Epidemiology & Community Health, School of Public Health, University of Minnesota, Minneapolis, MN 55454, USA

**Keywords:** phthalates, hypertension, pregnancy, blood pressure, preeclampsia

## Abstract

The purpose of this study was to determine whether maternal urinary phthalate metabolite concentrations are associated with the development of higher blood pressure or pregnancy-induced hypertension (PIH). Participants were women without chronic hypertension who enrolled in The Infant Development and the Environment Study, a prospective pregnancy cohort conducted at four U.S. academic medical centers from 2010–2012. Prenatal records were reviewed to obtain blood pressure measurements and diagnoses of PIH (gestational hypertension, preeclampsia, eclampsia, and HELLP syndrome, defined as hemolysis, elevated liver enzymes, and low platelet count). Complete-case analyses used multivariable linear and logistic regression for analysis of blood pressure measurements and PIH diagnoses, respectively. In the final dataset (N = 668), higher concentrations of first-trimester monoethyl phthalate (MEP) and mono-3-carboxypropyl phthalate (MCPP) and third-trimester mono-isobutyl phthalate (MiBP) were significantly associated with a medical chart diagnosis of PIH. First-trimester mono-n-butyl phthalate (MBP) and MEP along with the sum of di-(2-ethylhexyl) phthalate metabolites (∑DEHP) were each associated with increased systolic blood pressure across pregnancy. In conclusion, several phthalate metabolite concentrations were significantly associated with PIH and greater increases in systolic blood pressure across pregnancy.

## 1. Introduction

Hypertensive disease in pregnancy represents a major contributor to maternal morbidity and mortality in the U.S. and worldwide, and the incidence of these diseases is increasing [1,2,3]. The etiology of this rise is likely multifactorial, though older age at parturition, higher rates of obesity, and number of comorbidities appear to play key roles [4,5]. There is evidence that environmental factors, such as endocrine disrupting chemicals, including phthalates, may play a role as well.

Phthalates are chemicals that are used to increase the flexibility of plastics and are ubiquitous in the personal care product and food packaging industries [6]. Compared to men, adult women have higher urinary concentrations of specific phthalate metabolites that are frequently used in hygiene and cosmetic products [7]. Several phthalates are known endocrine disruptors [8,9,10,11,12,13], and there is burgeoning literature on the role of phthalates in hypertensive disorders. In the National Health and Nutrition Examination Survey (NHANES), higher concentrations of urinary mono-benzyl phthalate (MBzP), monobutyl phthalate (MBP), mono-n-methyl phthalate, mono-2-ethyl-5-oxohexyl phthalate (a metabolite of DEHP), and di-(2-ethylhexyl) terephthalate (DEHTP) metabolites were associated with high blood pressure in adult women [14]. Growing evidence has seen an association in children as well [15,16]. In cross-sectional work in children, the urinary sum of di-(2-ethylhexyl) phthalate metabolites (∑DEHP), mono-2-ethylhexyl phthalate (MEHP), and MBP metabolite concentrations were associated with elevated systolic blood pressure, and monoethyl phthalate (MEP) concentrations were associated with overall blood pressure that was greater than the 90th percentile in children aged 6–19 [16]. The mechanisms underlying this relationship may include direct effects on increased arterial tone via oxidative stress and endothelial injury or indirect effects via microvascular changes caused by insulin resistance [17,18,19].

There is growing literature indicating an association between maternal urinary phthalate concentrations and hypertensive disease in pregnancy [20], which may be mediated by trophoblastic dysfunction and subsequent abnormal blood vessel development at the maternal-placental interface. DEHP metabolites and mono-3-carboxypropyl phthalate (MCPP) have been linked to higher concentrations of serum pregnancy-associated plasma protein A (PAPP-A), a marker of placental function that is elevated in individuals with pregnancy-induced hypertension (PIH) [21], and urinary DEHP metabolites are inversely associated with maternal serum placental growth factor (PlGF), a biomarker of angiogenesis and, indirectly, placental function [21,22,23,24]. One prospective cohort study found that second-trimester maternal urinary MBzP concentrations were associated with elevations in diastolic blood pressure as well as the development of pregnancy-induced hypertensive disease [25]. A subsequent pregnancy cohort did not corroborate the association of MBzP but did report associations between other phthalate metabolites (MEHP, ∑DEHP, MCPP, and MEP) and a diagnosis of preeclampsia [26].

This study aims to further elucidate the role of maternal urinary phthalate metabolites in pregnancy on blood pressure in the first and third trimesters, increases in blood pressure over the course of pregnancy, and the development of PIH. We used data from a multi-center observational pregnancy cohort study. We hypothesized that first-trimester maternal urinary phthalate concentrations would be associated with greater increases in blood pressure, higher maternal blood pressure in the third trimester, and increased odds of PIH diagnosis after the urine sample.

## 2. Materials and Methods

### 2.1. Study Participants

The Infant Development and the Environment Study (TIDES) is a prospective cohort study of pregnant women recruited at four U.S. academic medical center sites, including the University of Minnesota, the University of California in San Francisco, the University of Washington, and the University of Rochester, between 2010 and 2012. Inclusion criteria included: gestational age less than 13 weeks, age of at least 18-years-old, able to read and write in English, no medical concerns for early pregnancy loss, and planning to deliver at the study site’s hospital. Participants with a diagnosis of chronic hypertension were excluded from the main analysis of this study but were included in the sensitivity analyses. Participants were asked to provide urine samples each trimester; for this study, only the first- and third-trimester phthalate values were measured. The women consented to the release of their pregnancy medical records for data abstraction. The primary aim of the prenatal TIDES was to determine the association between phthalates and anogenital distance; therefore, only pregnancies resulting in livebirths had their phthalates evaluated. Therefore, this study only includes women who delivered a livebirth. The study was approved by each site’s Institutional Review Board prior to the start of the study, and all participants signed written informed consent. Data abstraction from maternal medical records ascertained pregnancy information including maternal blood pressure values during the first- and third-trimester visits, ultrasound reports, pregnancy complication diagnoses, and details regarding delivery course and outcome.

### 2.2. Phthalate Metabolites in Maternal Urine

Maternal urine samples were collected in phthalate-free polypropylene cups, and all storage materials were phthalate-free. Specific gravity (SpG) was measured within 30 min of collection using a hand-held refractometer following calibration with de-ionized water. All samples were then stored at −80 degrees Celsius before the shipment of all of the samples to one of two laboratories: (1) the Division of Laboratory Sciences, National Center for Environmental Health, Center for Disease Control and Prevention (CDC) and (2) the Environmental Health Laboratory at the University of Washington (UW). Urine samples from the first trimester were then analyzed at each laboratory. All third-trimester urine samples were analyzed at the CDC. The CDC used enzymatic deconjugation of the metabolites from their glucuronidated form, automated online solid-phase extraction, separation with high performance liquid chromatography, and detection by means of isotope-dilution tandem mass spectrometry [27]. The UW technique included the enzymatic deconjugation of the metabolites from their glucuronidated form and automated online solid-phase extraction coupled with reversed high-performance liquid chromatography-electrospray ionization-tandem mass spectrometry (HPLC-ESI-MS/MS) to quantify the simple monoesters in the urine [28]. At both laboratories, procedure blanks were run with each batch of samples, and isotopically labeled internal standards were used along with conjugated internal standards to increase the precision and accuracy of the measurements. Travel blanks were collected at each study site at the beginning, middle, and end of the sample collection period and were analyzed with the participants’ samples. Limits of detection (LOD) were somewhat lower at the CDC lab compared to the UW lab. For the statistical analyses, values below the LOD from the first trimester (n = 631) and the first batch of the third trimester (n = 142) were assigned the LOD value divided by the square root of 2, as has been recommended when data are not highly skewed [29]. In the statistical analysis of the values below the LOD from the second batch of the third trimester (n = 493), the machine-read value was used as dictated by an update in CDC protocols. Most of the women in the TIDES pregnancy cohort had their urine samples from the first trimester analyzed for eight phthalate metabolites, and 10 phthalate metabolites were analyzed from the third-trimester samples (Table S2).

### 2.3. Medical Chart Abstraction

Maternal medical records were reviewed retrospectively by trained research staff to determine PIH diagnosis at any time during pregnancy and blood pressure measurements associated with the date of each study visit at which urine was collected (one visit each in the first and third trimesters). Any participant with a chronic hypertension diagnosis in the medical chart was excluded from the main analysis. Chronic hypertension diagnoses were identified via three mechanisms: (1) by ICD code during the pregnancy of interest, (2) as indicated by a provider’s note during the pregnancy of interest, or (3) if the participant reported taking antihypertensive medications every day for a week on their first-trimester survey. When a blood pressure value was not available at the date of urine collection, the subsequent visit’s blood pressure value was used. For visits with multiple blood pressure measurements taken, the last blood pressure measurement obtained on that date was used. This was to ensure an accurate resting blood pressure measurement and not a blood pressure measurement that was reflective of the patient’s exertion during their walk into the clinic.

PIH was defined as a diagnosis of at least one of the following as denoted by ICD codes or by providers’ notes in the medical record: gestational hypertension (ICD10 O13), pre-eclampsia with or without severe features or HELLP (hemolysis, elevated liver enzymes, and low platelet) syndrome (ICD9 642.4, ICD9 642.5, ICD10 O14), or eclampsia (ICD9 642.6, ICD10 O15; Table S1). The date of each diagnosis was recorded, and we defined a PIH case as a diagnosis made on or after the date of the relevant urine sample (see Section 2.4). For quality control, a secondary reviewer at each study site reviewed at least every tenth chart. If the findings were discordant, the records were jointly reviewed until consensus.

In addition to our PIH definition, we were interested in determining whether early phthalate exposure was associated with hypertension during pregnancy using the recently developed definition of hypertension in non-pregnant adults from the American College of Cardiology (ACC) and the American Heart Association (AHA), which is defined as systolic blood pressure (SBP) ≥ 130 mmHg or diastolic blood pressure (DBP) ≥ 80 mmHg [30]. This differs from the traditional definition of hypertension and the current definition of pregnancy-induced hypertension of SBP ≥ 140 mmHg or DBP ≥ 90 mmHg. Currently, the new ACC/AHA definition is not used for PIH diagnoses, as all outcome data are based on the traditional definition, but as research evaluates which is the most appropriate blood pressure cut-off, this definition may change. Therefore, we performed an additional analysis using this newer definition.

### 2.4. Statistical Methods

Phthalate metabolite concentrations (Table S2) were natural-log-transformed to normalize right-skewed distributions and were adjusted for urine dilution using the following formula: Pc = P × [(SpGmed-1)/(SpG-1)], where SpG is the specific gravity of the individual urine sample, Pc is the SpG-corrected phthalate concentration (ng/mL), P is the observed phthalate concentration (ng/mL), and SpGmed is the median SpG for all TIDES samples at that timepoint [31]. Table S3 summarizes the specific gravity-adjusted phthalate distributions and proportions above the limit of detection. Molar sums were computed by summing the phthalate components of the parent compound with each component divided by its molecular weight. That is, ∑DEHP = ((MEHP/278) + (MEHHP/294) + (MECPP/308) + (MEOHP/292)) × 1000 nmol/L and ∑DEHTP = ((MEHHTP/294) + (MECPTP/312)) × 1000 nmol/L.

**Figure 1:**
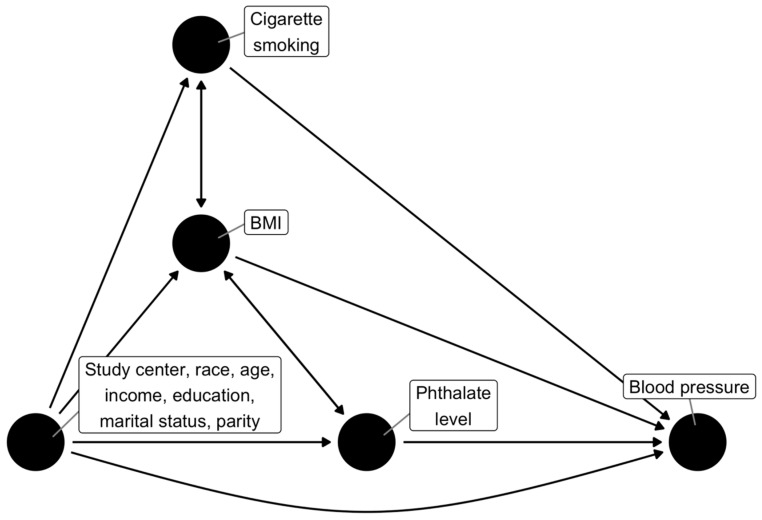
Hypothesized causal DAG for analysis of phthalate level and PIH. Double-headed arrows indicate a latent confounder.

Control variables were selected through the use of a directed acyclic graph (DAG; Figure 1), which was constructed based on previous literature [32,33,34]. If the causal diagram in Figure 1 is correct, the set of variables needed to control for confounding is: study center, race, age, household income, highest level of education, marital status, parity, pre-pregnancy BMI, and cigarette smoking in the first trimester. Age and pre-pregnancy BMI were analyzed as continuous variables, whereas the remaining covariates were categorical as listed in Table 1. To increase precision, we also included gestational age at blood pressure measurement as a control variable in the SBP and DBP models.

After assessing linearity through partial regression plots and partial residual plots, we used multivariable linear regression to analyze the continuous outcomes of interest, SBP and DBP, in both the first and third trimesters. We examined the association between blood pressure change (i.e., trimester difference in SBP, difference in DBP) with first-trimester phthalates as well as the association of third-trimester blood pressures with (a) first-trimester, (b) third-trimester, and (c) the average of the first- and third-trimester maternal urinary phthalate metabolite concentrations. The average value acts as a surrogate for what may be average exposure across the span on the pregnancy, given that the half-life of most phthalate metabolites is <24 h [35,36].

We also considered two dichotomous outcomes: (1) diagnosis of PIH based on medical chart abstraction and (2) the 2017 ACC/AHA hypertension definition of SBP ≥ 130 mmHg or DBP ≥ 80 mmHg [30]. We used logistic regression to measure the association of these outcomes with the first-trimester, third-trimester, and the average of the first- and third-trimester phthalates. We defined a case as a diagnosis made or high blood pressure obtained on or after the date of the relevant urine sample. For the phthalate average, we defined a case as a diagnosis made or high blood pressure obtained on or after the date of the third-trimester urine sample.

Our final analysis set only included women who had data on all variables (complete-case analyses). Because we analyzed complete cases only, the model sample sizes ranged from 517 to 614, depending on the outcome variable, except for those considering the ∑DEHTP metabolite concentration, which had complete-case sample sizes of 436 to 472. All of the analyses were conducted using R software, Version 3.6.2.

### 2.5. Sensitivity Analyses

We performed several sensitivity analyses, including a series to assess for potential bias due to missing data. First, we assessed whether a missing urinary metabolite value was associated with any key covariates. Second, we compared covariates between groups with and without blood pressure measurements, which we restricted to measurements on or after the date of the respective trimester’s urine sample. Third, we estimated the relationship between hypertension during pregnancy and missing covariates, which were primarily provided through surveys.

We also explored the extent to which this study’s phthalate metabolites associated differently when we included the women who had chronic hypertension, both including and excluding those who reported antihypertensive medication use in the first trimester. Finally, we evaluated whether the inclusion of the ICD9 code 642.3, “transient hypertension,” changed our results, as this diagnostic code included both individuals with actual transient hypertension (not a hypertensive disease of pregnancy) and gestational hypertension, which is included in the PIH definition.

## 3. Results

### 3.1. Demographic Characteristics

Live births were recorded for 805 TIDES participants, 67 of whom were excluded from our analyses due to chronic hypertension. Of the resultant 738 participants, 668 had complete data on race, age at delivery, education, marital status, income, smoking, pre-pregnancy BMI, and parity, and these women were included as participants in the current analyses. First- and third-trimester urinary phthalate metabolite concentrations for mono-isobutyl phthalate (MiBP), MEP, MBzP, MBP, MEHP, MCPP, and ∑DEHP were available for 631 and 635 participants, respectively. In addition, third-trimester concentrations for ∑DEHTP were available for 493 participants. A total of 539 participants had first-trimester blood pressure values available at or after the time of the first-trimester urine sample, and 566 participants had third-trimester blood pressure values available at or after the time of the third-trimester urine sample.

Most of the sample was white (68.7%), had graduated college (73.6%), was married or living as married (83.4%), had a household income of at least USD 45,000 (64.3%), and did not smoke cigarettes (92.3%) (Table 1). Of the 45 women defined as having PIH, nearly all (n = 43) were diagnosed with pre-eclampsia, with the majority at the Minnesota (n = 17) or Rochester (n = 19) study centers. There was no significant difference in PIH diagnosis from the medical record based on the laboratory site or laboratory batch of urinary phthalate analysis.

### 3.2. PIH and Phthalate Associations

After adjusting for covariates, higher first-trimester maternal urinary concentrations of monoethyl phthalate (MEP) and mono-3-carboxypropyl phthalate (MCPP) were significantly associated with the diagnosis of PIH (adjusted OR 1.4, CI 1.09–1.79, *p* = 0.01 and adjusted OR 1.34, CI 1.05–1.7, *p* = 0.02; respectively for each ln-unit increase), as was the third-trimester concentration of monoisobutyl phthalate (MiBP) (adjusted OR 1.5, CI 1.01–2.22, *p* = 0.04). Higher averages of the first- and third-trimester maternal urinary phthalate metabolite concentrations of MEP and MiBP were also significantly associated with PIH diagnosis (adjusted OR 1.36, CI 1.03–1.79, *p* = 0.03 and adjusted OR 1.8, 1.09–2.97, *p* = 0.02; respectively; Table 2).

### 3.3. Hypertension by the New ACC/AHA Guidelines

We did not observe any significant associations between phthalate metabolites and hypertension diagnosis under the new ACC/AHA guidelines (Table 2).

### 3.4. Systolic and Diastolic Blood Pressure

After controlling for covariates, first-trimester maternal urinary concentrations MBP, MEP, and ∑DEHP were positively associated with the difference between first- and third-trimester SBP (β 1.65, CI 0.3–2.99, *p* = 0.02; β 0.96, CI 0.11–1.8, *p* = 0.03; and β 1.52, CI 0.17–2.86, *p* = 0.03, respectively); average MiBP was positively associated with third-trimester SBP (β 1.44, CI 0.05–2.84, *p* = 0.04). We also observed a significantly negative association between first-trimester MEP and first-trimester SBP (β −0.83, CI −1.54–−0.12, *p* = 0.02).

We observed no significant associations between the third-trimester maternal urinary phthalate concentrations and systolic or diastolic blood pressure, and no significant associations between the phthalate concentrations and DBP at either trimester were found (Table 3).

### 3.5. Sensitivity Analyses

For each trimester, we compared the distributions of the demographic and behavioral covariates shown in Table 1 between the participants with and without urine samples. The only significant difference was by study center; participants at the University of Washington were more likely to be missing third-trimester phthalate concentrations. The UCSF study center had a significantly higher proportion of missing first-trimester blood pressure data than the other three centers, with about 37% of participants missing data compared to less than 10% at each of the other centers. The UCSF participants were also significantly older, had higher income, and were more likely to be non-Black and married than the other sites pooled; therefore, missing first-trimester blood pressure data was significantly associated with these characteristics. In the third trimester, UCSF again had a significantly higher proportion of missing blood pressure data but by a smaller margin, with about 25% of participants missing data compared to 19% at UW, the next highest. Parity, which had a significantly different distribution at UCSF compared to the pooled other centers, was significantly associated with missing third-trimester blood pressure data. Only the 2017 ACC/ACA definition of hypertension was significantly associated with an indicator for any missing covariate data; missingness was associated with greater odds of hypertension.

In the sensitivity analyses that included the 67 participants with chronic hypertension, certain urinary phthalate concentrations, MEHP, MEP, and ∑DEHP were associated with pregnancy-induced hypertension diagnoses; MBzP was associated with third-trimester SBP; and MBP and MEP were associated with trimester differences in SBP. When only the participants that reported taking antihypertensive medication in the first trimester were excluded, only the MEP and ∑DEHP urinary metabolites were associated with pregnancy-induced hypertension diagnoses, and MBP, MEP, and ∑DEHP were associated with SBP difference (Tables S4 and S5). Results did not vary based on the inclusion of ICD9 code 642.3, “transient hypertension”.

## 4. Discussion

In our prospective pregnancy cohort, we found evidence that certain phthalate metabolites were associated with a higher estimated risk of PIH as well as higher blood pressure over the course of the pregnancy. The significant associations between phthalate concentrations and systolic blood pressure difference in our analyses ranged from about 1–1.65 mmHg per unit increase in natural-log-transformed phthalate concentration (ng/mL or nmol/L) between the third and first trimesters. For the most part, this is not a clinically meaningful change in blood pressure, and it suggests that women may be vulnerable to environmental insults that in totality of all of her other risks may place her at greater risk of a hypertensive disorder in pregnancy. Notably, we believe our study is the first to observe significantly increased odds of PIH diagnosis as well as increased systolic blood pressure over the pregnancy, with higher first-trimester MEP levels being observed. This finding is particularly interesting because of its consistency across these two blood pressure outcomes.

### 4.1. Pregnancy-Induced Hypertension

When we examined whether the timing and level of phthalate metabolites in pregnancy had an effect on PIH diagnosis in our multicenter cohort, we observed higher concentrations of first-trimester MEP and MCPP, and higher concentrations of third-trimester MiBP were significantly associated with a medical chart diagnosis of PIH. In addition, when averaged over the pregnancy, the MEP and MiBP metabolites were also associated with PIH diagnosis. This is consistent with prior studies that showed that increased urinary MCPP was associated with PIH and with serum concentrations of PAPP-A, a marker associated with PIH [21,26]. Interestingly, MCPP has not been associated with hypertension in nonpregnant women. However, unlike a previous report by Cantonwine [26], we found MiBP to be associated with diagnosis of PIH, whereas Cantonwine noted a negative association between mid-pregnancy urinary MiBP concentrations and preeclampsia.

Similar to two of the three other pregnancy cohort studies on this topic, we did not find an association between a ∑DEHP-induced diagnosis of PIH or incident maternal blood pressure values, though we did find an association with the increase in SBP [24,25,26]. Interestingly, the study by Philips et al. did note a relationship between the ∑DEHP and early pregnancy soluble fms-tyrosene kinase (sFit-1)/placental growth factor (PlGF) ratios, the elevation of which is highly predictive of preeclampsia [24]. The two studies that have previously evaluated MBzP in association with PIH had conflicting findings—our results indicate no association with PIH [25,26]. The relationship we identified between maternal urinary MBP and blood pressure is inconsistent with prior pregnancy cohorts but is consistent with data on hypertension in children [16,26].

Our primary aim was to determine the odds of incident PIH, but we also explored the extent to which these phthalate metabolites associated differently in the subsample of pregnant women who had chronic hypertension and who were excluded from our primary analyses. In the sensitivity analyses that included the 67 participants with chronic hypertension, certain urinary phthalate concentrations, including MEHP, MEP, and ∑DEHP, were associated with PIH diagnoses (Table S4). The association with MEHP is interesting because this is consistent with findings from the two other studies that included those with chronic hypertension in the analyses [23,24]. Further sensitivity analyses only excluded the participants that reported taking antihypertensives in the first trimester to evaluate for potential interactions between the medications themselves and phthalate effects (Tables S4 and S5). When we considered the statistical interactions between phthalate exposure and antihypertensive use, we found evidence that the medications indeed modified the association between phthalate metabolites and PIH or blood pressure. The interaction terms with antihypertensive medication use were highly significant (*p* < 0.01) for MBzP, MEHP, MCPP, MiBP, ∑DEHP, and ∑DEHTP. The main effects of the phthalates were not significant in these models, though, and the direction (positive or negative) of the association between phthalate exposure and blood pressure or PIH among women on antihypertensives was not consistent.

### 4.2. Blood Pressure over the Pregnancy

We were also interested in examining the timing and the phthalate level on changes in blood pressure over the course of the pregnancy. In doing so, we observed that only first-trimester exposures were significantly associated with increased systolic blood pressure between the first and third trimesters. Specifically, we found that MBP, MEP, and the sum of the di-(2-ethylhexyl) phthalate metabolites (∑DEHP) were each associated with a significant but slight increase in systolic blood pressure across pregnancy. There were no significant findings for changes in blood pressure (systolic or diastolic) when examining any of the third-trimester or averaged phthalate metabolite levels.

### 4.3. Strengths and Limitations

The strengths of this study include the large sample size, the diversity of the participants from across the U.S., and its multisite design. There are three previously published studies of PIH in relation to phthalates in pregnancy. The Werner et al. [25] study included those with chronic hypertension, which is a known risk factor for the development of PIH and therefore may confound the conclusions drawn from this cohort. Both of the other studies, by Cantonwine et al. [26] and Philips et al. [24], excluded those with chronic hypertension, similar to our study. Cantonwine et al. [26] and Werner et al. used smaller cohorts, while Philips et al. had a larger cohort with 1233 women. Cantonwine et al. included three academic centers in urban east coast cities, while Werner et al. represented a single geographic area, thus limiting generalizability.

Some limitations in our study design may affect the interpretation of our results, such as the variation in the detailed documentation inherent to the chart review and the missing urine samples and blood pressure values in some cases. However, our sensitivity analyses showed little effect of these missing values. Single spot urine samples may poorly reflect phthalate metabolite concentrations across a whole trimester due to their short half-life. On the other hand, the average concentration might serve as a proxy for typical phthalate exposure during pregnancy. In fact, we estimated larger coefficients for the average than for the concentration at either trimester in the analyses for MiBP, which could indicate a broad window of susceptibility during pregnancy. Future studies should consider taking more frequent measurements of both phthalate metabolite concentration and blood pressure to confirm whether there are critical windows for phthalate exposure with regard to hypertension in pregnancy. Lastly, we excluded ICD9 code 642.3, “transient hypertension,” which includes some participants diagnosed with gestational hypertension. This decision was made because otherwise, individuals with a diagnosis of true transient hypertension in pregnancy, which is not considered a hypertensive disease in pregnancy, might have been included and thus would have affected the strength of our findings. However, in the sensitivity analysis, our results were relatively unchanged by inclusion of this ICD9 code. This could be because transient hypertension is often the first diagnosis prior to a diagnosis of full-fledged pregnancy-induced hypertension due to the nature of the disease. A transient hypertension diagnosis would be met if a single measurement had SBP ≥ 140 mmHg or DBP ≥ 90 mmHg, whereas to meet the criteria for gestational hypertension, a patient must have two separate elevated blood pressures more than four hours apart.

## 5. Conclusions

In conclusion, our multicenter cohort found that the urinary MEP, MCPP, and MiBP levels of pregnant women were positively associated with PIH, and that pregnant women’s first-trimester urinary MBP, MEP, and ∑DEHP were associated with an increase in systolic blood pressure from the first to third trimester. Our results add to the body of literature in support of the association between phthalates and elevations in blood pressure in pregnant women. Given the significant maternal and fetal morbidity and mortality associated with pregnancy-induced hypertension, the identification of modifiable risk factors that are present at a population-level, such as environmental exposure to phthalates, has serious public health implications. The physiologic pathway that may explain this finding has yet to determined, and therefore, more research on the temporal pathway of the timing of phthalate exposure on the onset of higher blood pressure in pregnancy is warranted.

## Figures and Tables

**Table 1:** Demographics and pregnancy characteristics of 738 pregnant women from The Infant Development and the Environment Study (TIDES), 2010–2012.

Variable	N (%)	Mean +/− SD
All Participants	738	
Study Center		
University of California in San Francisco	183 (24.8)	
University of Minnesota	198 (26.8)	
University of Rochester	204 (27.6)	
University of Washington	151 (20.5)	
Gestational Age at First-Trimester Survey (weeks)		12.75 +/− 3.87
Missing	9 (1.2)	
Maternal Race		
White	507 (68.7)	
Black	93 (12.6)	
Asian, Native American, multiple race, other	122 (16.5)	
Missing	16 (2.2)	
Age at Delivery		31.00 +/− 5.52
Missing	3 (0.0)	
Highest Education		
Some high school or lower	59 (8.0)	
Graduated high school	43 (5.8)	
Some college or technical school	83 (11.2)	
Graduated college or technical school	226 (30.6)	
Some graduate work or graduate degree	317 (43.0)	
Missing	10 (1.4)	
Marital Status		
Married	515 (69.8)	
Living as married	100 (13.6)	
Separated, divorced, single	118 (16.0)	
Missing	5 (0.7)	
Household Income		
<USD 15,000	103 (14.2)	
USD 15–45,000	131 (17.8)	
USD 45–75,000	133 (18.0)	
USD 75,000+	342 (46.3)	
Missing	29 (3.9)	
Cigarette Smoking in First Trimester		
Yes	42 (5.7)	
No	681 (92.3)	
Missing	15 (2.0)	
Pre-pregnancy BMI		25.10 +/− 5.73
Missing	13 (1.8)	
Parity		
0	367 (49.7)	
1	228 (30.9)	
2+	102 (13.8)	
Missing	41 (5.6)	

**Table 2:** Adjusted odds ratios (95% confidence interval, CI) of a diagnosis of hypertensive disease by using the medical record or the 2017 ACC/AHA definition by first- (T1), third- (T3), and average maternal urinary concentrations of specific phthalate metabolites in pregnant women in The Infant Development and the Environment Study (TIDES), 2010–2012.

	Pregnancy-Induced Hypertension by Medical Record		Hypertension by 2017 ACC/AHA Definition	
ln (Phthalate)	aOR (95% CI)	N	aOR (95% CI)	N
T1 MBzP	1.06 (0.72–1.55)	609	1.12 (0.89–1.43)	588
T3 MBzP	1.06 (0.76–1.48)	614	1.15 (0.92–1.44)	567
Average MBzP	1.1 (0.71–1.7)	585	1.27 (0.95–1.7)	542
T1 MBP	1.21 (0.79–1.84)	609	0.91 (0.7–1.19)	588
T3 MBP	1.15 (0.78–1.69)	614	1.25 (0.97–1.61)	567
Average MBP	1.27 (0.78–2.08)	585	1.38 (0.98–1.94)	542
T1 MEHP	1.21 (0.83–1.75)	609	0.91 (0.7–1.17)	588
T3 MEHP	1.16 (0.76–1.75)	614	0.85 (0.64–1.12)	567
Average MEHP	1.25 (0.76–2.06)	585	0.79 (0.55–1.15)	542
T1 MEP	**1.4 (1.09–1.79**)	609	1.01 (0.85–1.19)	588
T3 MEP	1.22 (0.95–1.55)	614	1.06 (0.9–1.26)	567
Average MEP	**1.36 (1.03–1.79)**	585	1.04 (0.87–1.26)	542
T1 MCPP	**1.34 (1.05–1.7)**	609	1.01 (0.85–1.2)	588
T3 MCPP	0.95 (0.7–1.3)	614	1.05 (0.86–1.27)	567
Average MCPP	1.03 (0.75–1.42)	585	1.03 (0.83–1.28)	542
T1 MiBP	1.5 (0.95–2.36)	609	0.88 (0.66–1.17)	588
T3 MiBP	**1.5 (1.01–2.22)**	614	1.06 (0.82–1.38)	567
Average MiBP	**1.8 (1.09–2.97)**	585	1.16 (0.83–1.63)	542
T1 ∑DEHP	1.32 (0.9–1.95)	609	1 (0.77–1.32)	588
T3 ∑DEHP	1.34 (0.87–2.07)	614	1.05 (0.77–1.45)	567
Average ∑DEHP	1.42 (0.87–2.31)	585	1.08 (0.75–1.56)	542
T3 ∑DEHTP	1.02 (0.73–1.43)	477	1.14 (0.93–1.39)	460

Note: Models were adjusted for study center, race, age at delivery, household income, highest level of education, marital status, cigarette smoking in the first trimester, pre-pregnancy BMI, and parity. Significant results are shown in bold.

**Table 3:** Multivariable linear regression coefficients for first- (T1) and third-trimester (T3) blood presScheme 2010–2012.

	Estimated Phthalate Coefficient (95% CI)					
ln (Phthalate)	T1 SBP	T1 DBP	T3 SBP	T3 DBP	T3 SBP-T1 SBP	T3 DBP-T1 DBP
T1 MBzP	−0.41 (−1.41–0.6)	0.24 (−0.46–0.94)	0.88 (−0.18–1.94)	0.57 (−0.12–1.26)	1.07 (−0.12–2.26)	0.28 (−0.51–1.08)
T3 MBzP			0.57 (−0.33–1.47)	−0.11 (−0.71–0.49)		
Average MBzP			0.86 (−0.32–2.05)	0.07 (−0.7–0.83)		
T1 MBP	−0.78 (−1.91–0.35)	0.34 (−0.45–1.13)	0.92 (−0.26–2.1)	0.26 (−0.51–1.03)	**1.65 (0.3–2.99)**	−0.14 (−1.04–0.76)
T3 MBP			0.46 (−0.6–1.53)	0.02 (−0.68–0.73)		
Average MBP			1.04 (−0.41–2.49)	0.16 (−0.78–1.1)		
T1 MEHP	−0.31 (−1.37–0.74)	0.17 (−0.57–0.9)	−0.03 (−1.09–1.03)	−0.28 (−0.97–0.41)	0.31 (−0.94–1.55)	−0.8 (−1.62–0.03)
T3 MEHP			−0.41 (−1.48–0.67)	−0.37 (−1.09–0.35)		
Average MEHP			−0.25 (−1.52–1.03)	−0.28 (−1.11–0.54)		
T1 MEP	**−0.83 (−1.54–−0.12)**	−0.35 (−0.85–0.15)	0.24 (−0.51–1)	−0.2 (−0.7–0.29)	**0.96 (0.11–1.8)**	0.07 (−0.5–0.63)
T3 MEP			0.38 (−0.29–1.05)	−0.22 (−0.67–0.23)		
Average MEP			0.38 (−0.39–1.15)	−0.17 (−0.66–0.33)		
T1 MCPP	−0.03 (−0.76–0.7)	0.21 (−0.3–0.72)	0.65 (−0.14–1.44)	0.18 (−0.33–0.7)	0.64 (−0.24–1.52)	0.04 (−0.55–0.62)
T3 MCPP			0.37 (−0.39–1.13)	0.2 (−0.31–0.7)		
Average MCPP			0.61 (−0.26–1.47)	0.29 (−0.27–0.85)		
T1 MiBP	−0.41 (−1.61–0.79)	−0.21 (−1.05–0.63)	0.88 (−0.36–2.12)	0.23 (−0.58–1.04)	1.2 (−0.24–2.63)	0.48 (−0.49–1.44)
T3 MiBP			0.66 (−0.43–1.74)	−0.09 (−0.81–0.63)		
Average MiBP			**1.44 (0.05–2.84)**	0.34 (−0.57–1.25)		
T1 ∑DEHP	−0.54 (−1.68–0.6)	0.04 (−0.76–0.83)	0.73 (−0.43–1.88)	0.13 (−0.63–0.89)	**1.52 (0.17–2.86)**	−0.2 (−1.1–0.7)
T3 ∑DEHP			0.2 (−1.06–1.46)	0.02 (−0.82–0.86)		
Average ∑DEHP			0.59 (−0.79–1.97)	0.14 (−0.75–1.04)		
T3 ∑DEHTP			0.61 (−0.26–1.48)	0.31 (−0.27–0.89)		

Note: Models were adjusted for study center, race, age at delivery, household income, highest level of education, marital status, cigarette smoking in the first trimester, pre-pregnancy BMI, parity, and gestational age. Significant results are shown in bold.

## Supplementary Materials

The following are available online at https://www.mdpi.com/article/10.3390/ijerph182010627/s1, Table S1: ICD9/10 codes used for pregnancy-induced hypertension (PIH) definition, Table S2: Phthalate metabolites analyzed and corresponding parent compounds, Table S3: Summary of specific gravity-adjusted phthalate distributions, Table S4: Adjusted odds ratios of a diagnosis of hypertensive disease in pregnancy based on first-, third-, and average maternal urinary concentrations of specific phthalate metabolites, Table S5: Multivariable linear regression coefficients for first-, third-, and average maternal urinary concentrations of specific phthalate metabolites as predictors of third-trimester blood pressures.
