# Supplementary material for: First- and Third-Trimester Urinary Phthalate Metabolites in the Development of Hypertensive Diseases of Pregnancy
Source: Int J Environ Res Public Health. 2021 Oct 11;18(20):10627. doi: 10.3390/ijerph182010627 (PMC8536149; doi:10.3390/ijerph182010627)
Supplement: Supplementary file 1 [file ijerph-18-10627-s001.zip › ijerph-1385738-supplementary.pdf]

International Journal of
Environmental Research and Public Health

Supplementary Materials

Table S1. ICD9/10 codes used for pregnancy-induced hypertension (PIH) definition.

Hypertension in Pregnancy Diagnoses	ICD9 & ICD10 Codes
Gestational hypertension	ICD10 O13
Pre-eclampsia without severe features	ICD9 642.4, ICD10 O14.0, ICD10 O14.9
Pre-eclampsia with severe features	ICD9 642.5, ICD10 O14.1
Eclampsia	ICD9 642.6, ICD10 O15
Hemolysis, elevated liver enzymes, low platelets (HELLP) syndrome	ICD9 642.5, ICD10 O14.2

Table S2. Phthalate metabolites analyzed and corresponding parent compounds. Metabolites (bolded) of the parent compounds (in italics) were measured in urine samples.

Phthalates and Plasticizers Metabolites Panel	Parent Compound
Mono(2-ethyl-5-hydroxyhexyl) terephthalate	*Di(2-ethylhexyl) terephthalate (DEHTP)*
Mono(2-ethyl-5-carboxypentyl) terephthalate	
Mono-2-ethyl-5-carboxypentyl phthalate	*Di(2-ethylhexyl) phthalate (DEHP)*
Mono-2-ethyl-5-hydroxyhexyl phthalate	
Mono-2-ethyl-5-oxohexyl phthalate	
**Mono-2-ethylhexyl phthalate (MEHP)**	
**Mono-3-carboxypropyl phthalate (MCPP)**	Di-n-octyl phthalate (DOP), other high molecular weight phthalates, DBP (minor)
**Mono-n-butyl phthalate (MBP)**	Di-n-butyl phthalate (DBP or DnBP)
Mono-3-hydroxybutyl phthalate	
**Monobenzyl phthalate (MBzP)**	Benzylbutyl phthalate (BBzP)
**Mono-isobutyl phthalate (MiBP)**	Di-iso-butyl phthalate (DiBP)
Mono-2-methyl-2-hydroxypropyl phthalate	
**Monoethyl phthalate (MEP)**	Diethyl phthalate (DEP)

Table S3. Summary of specific gravity-adjusted phthalate distributions.

	First Trimester				Third Trimester			
**Phthalate**	**% ≥ LOD**	**Mean**	**SD**	**Median**	**% ≥ LOD**	**Mean**	**SD**	**Median**
MBzP (ng/mL)	86.8	7.60	15.87	3.92	94.8	10.23	31.37	4.48
lnMBzP		1.42	1.03	1.37		1.53	1.18	*1.50*
MBP (ng/mL)	92.6	12.70	24.47	8.18	98.0	20.05	134.94	10.15
lnMBP		2.09	0.89	2.10		2.24	0.97	*2.32*
MEHP (ng/mL)	67.2	5.18	20.34	2.43	74.7	3.49	7.64	1.96
lnMEHP		0.92	0.93	0.89		0.66	0.97	*0.67*
MEP (ng/mL)	98.9	118.93	360.87	29.73	98.6	251.16	1834.69	32.10
lnMEP		*3.55*	*1.36*	*3.39*		*3.68*	*1.54*	*3.47*
MCPP (ng/mL)	75.1	9.47	43.22	2.05	87.1	8.62	33.12	1.83
lnMCPP		*0.92*	*1.30*	*0.72*		*0.81*	*1.34*	*0.60*

MiBP (ng/mL)	96.8	7.29	7.63	5.01	96.1	12.89	28.22	6.72
lnMiBP		*1.62*	*0.86*	*1.61*		*1.96*	*0.96*	*1.91*
∑DEHP (nmol/L)	See (a)	177.22	584.77	84.02	See (b)	143.76	284.09	89.23
ln∑DEHP		*4.52*	*0.86*	*4.43*		*4.51*	*0.82*	*4.49*
∑DEHTP (nmol/L)					See (c)	61.27	245.94	10.93

a) Molar sum of MEHP (67.2% ≥ LOD), MEHHP (97.6%), MECPP (97.8%), and MEOHP (96.5%); (b) Molar sum of MEHP (74.7% ≥ LOD), MEHHP (99.4%), MECPP (100%), and MEOHP (99.5%); (c) Molar sum of MEHHTP (53.4% ≥ LOD) and MECPTP (93.7%).

Table S4. Adjusted odds ratios of a diagnosis of hypertensive disease in pregnancy based on first-, third-, and average maternal urinary concentrations of specific phthalate metabolites.

	**All Participants**		**Excluding Those on Antihypertensives**		**Excluding Those with Chronic Hypertension (Main Analysis)**	
**ln(Phthalate)**	**PIH by Medical Record aOR (95% CI)**	**Hypertension by 2017 ACC/AHA Definition aOR (95% CI)**	**PIH by Medical Record aOR (95% CI)**	**Hypertension by 2017 ACC/AHA Definition aOR (95% CI)**	**PIH by Medical Record aOR (95% CI)**	**Hypertension by 2017 ACC/AHA Definition aOR (95% CI)**
T1 MBzP	1.08 (0.81–1.46)	1.07 (0.86–1.32)	1.1 (0.8–1.51)	1.08 (0.86–1.35)	1.06 (0.72–1.55)	1.12 (0.89–1.43)
T3 MBzP	1.25 (0.94–1.67)	1.16 (0.95–1.41)	1.12 (0.83–1.51)	1.15 (0.94–1.42)	1.06 (0.76–1.48)	1.15 (0.92–1.44)
Average MBzP	1.35 (0.95–1.93)	1.21 (0.94–1.55)	1.17 (0.8–1.69)	1.25 (0.96–1.63)	1.1 (0.71–1.7)	1.27 (0.95–1.7)
T1 MBP	0.99 (0.7–1.38)	0.88 (0.69–1.12)	1.04 (0.73–1.48)	0.87 (0.68–1.12)	1.21 (0.79–1.84)	0.91 (0.7–1.19)
T3 MBP	1.28 (0.93–1.76)	1.23 (0.98–1.54)	1.14 (0.81–1.59)	1.2 (0.95–1.52)	1.15 (0.78–1.69)	1.25 (0.97–1.61)
Average MBP	1.36 (0.91–2.03)	1.3 (0.96–1.75)	1.15 (0.74–1.78)	1.29 (0.94–1.77)	1.27 (0.78–2.08)	1.38 (0.98–1.94)
T1 MEHP	0.98 (0.71–1.35)	0.85 (0.67–1.08)	1.07 (0.76–1.49)	0.84 (0.66–1.07)	1.21 (0.83–1.75)	0.91 (0.7–1.17)
T3 MEHP	**1.44 (1.01–2.05)**	0.84 (0.66–1.08)	1.3 (0.9–1.87)	0.81 (0.62–1.05)	1.16 (0.76–1.75)	0.85 (0.64–1.12)
Average MEHP	1.39 (0.93–2.08)	0.77 (0.56–1.06)	1.33 (0.87–2.04)	0.76 (0.54–1.06)	1.25 (0.76–2.06)	0.79 (0.55–1.15)
T1 MEP	**1.37 (1.11–1.69)**	1.02 (0.88–1.19)	**1.38 (1.11–1.72)**	0.99 (0.85–1.16)	**1.4 (1.09–1.79)**	1.01 (0.85–1.19)
T3 MEP	**1.24 (1.01–1.52)**	1.05 (0.91–1.22)	**1.27 (1.02–1.56)**	1.08 (0.93–1.26)	1.22 (0.95–1.55)	1.06 (0.9–1.26)
Average MEP	**1.42 (1.13–1.79)**	1.05 (0.89–1.24)	**1.4 (1.1–1.78)**	1.05 (0.89–1.26)	**1.36 (1.03–1.79)**	1.04 (0.87–1.26)
T1 MCPP	1.07 (0.86–1.33)	0.99 (0.84–1.17)	1.15 (0.92–1.43)	1 (0.85–1.18)	**1.34 (1.05–1.7)**	1.01 (0.85–1.2)
T3 MCPP	1.04 (0.81–1.34)	1.01 (0.85–1.21)	1.03 (0.79–1.33)	1.03 (0.86–1.24)	0.95 (0.7–1.3)	1.05 (0.86–1.27)
Average MCPP	1.01 (0.77–1.34)	1 (0.82–1.22)	1.03 (0.77–1.36)	1.01 (0.82–1.23)	1.03 (0.75–1.42)	1.03 (0.83–1.28)
T1 MiBP	1.03 (0.72–1.46)	0.87 (0.68–1.13)	1.12 (0.77–1.62)	0.87 (0.66–1.13)	1.5 (0.95–2.36)	0.88 (0.66–1.17)
T3 MiBP	1.29 (0.92–1.81)	1.01 (0.79–1.28)	1.28 (0.9–1.82)	1.03 (0.8–1.31)	**1.5 (1.01–2.22)**	1.06 (0.82–1.38)
Average MiBP	1.44 (0.93–2.22)	1.04 (0.77–1.42)	1.41 (0.9–2.22)	1.1 (0.8–1.51)	**1.8 (1.09–2.97)**	1.16 (0.83–1.63)
T1 ∑DEHP	1.07 (0.76–1.49)	0.99 (0.78–1.27)	1.19 (0.84–1.68)	0.98 (0.76–1.26)	1.32 (0.9–1.95)	1 (0.77–1.32)
T3 ∑DEHP	**1.67 (1.15–2.41)**	1.07 (0.81–1.42)	**1.51 (1.02–2.22)**	1.04 (0.77–1.4)	1.34 (0.87–2.07)	1.05 (0.77–1.45)
Average ∑DEHP	**1.71 (1.15–2.55)**	1.12 (0.81–1.54)	**1.58 (1.04–2.41)**	1.09 (0.78–1.54)	1.42 (0.87–2.31)	1.08 (0.75–1.56)
T3 ∑DEHTP	0.99 (0.74–1.33)	1.07 (0.88–1.29)	1 (0.75–1.35)	1.09 (0.9–1.33)	1.02 (0.73–1.43)	1.14 (0.93–1.39)

Note: Models were adjusted for study center, race, age at delivery, household income, highest level of education, marital status, cigarette smoking in the first trimester, pre-pregnancy BMI, parity, and antihypertensive use (where applicable). PIH = pregnancy-induced hypertension. Significant results are shown in bold.

Table S5. Multivariable linear regression coefficients for first-, third-, and average maternal urinary concentrations of specific phthalate metabolites as predictors of third-trimester blood pressures. Models including all TIDES participants were adjusted for antihypertensive use.

**Estimated Phthalate Coefficient (95% CI)**

	All Participants		Excluding Those on Antihypertensives		Excluding Those with Chronic Hypertension (Main Analysis)	
**ln(Phthalate)**	**T3 SBP**	**T3 SBP-T1 SBP**	**T3 SBP**	**T3 SBP-T1 SBP**	**T3 SBP**	**T3 SBP-T1 SBP**
T1 MBzP	0.98 (−0.09–2.04)	1.14 (−0.07–2.36)	0.74 (−0.31–1.79)	1.08 (−0.11–2.26)	0.88 (−0.18–1.94)	1.07 (−0.12–2.26)
T3 MBzP	0.87 (−0.02–1.77)		0.67 (−0.22–1.55)		0.57 (−0.33–1.47)	
Average MBzP	**1.18 (0.01–2.35)**		0.83 (−0.32–1.99)		0.86 (−0.32–2.05)	
T1 MBP	0.71 (−0.49–1.9)	**1.39 (0–2.77)**	0.67 (−0.51–1.85)	**1.6 (0.25–2.96)**	0.92 (−0.26–2.1)	**1.65 (0.3–2.99)**
T3 MBP	0.75 (−0.29–1.8)		0.45 (−0.59–1.5)		0.46 (−0.6–1.53)	
Average MBP	1.25 (−0.16–2.67)		0.79 (−0.64–2.22)		1.04 (−0.41–2.49)	
T1 MEHP	−0.35 (−1.42–0.72)	0.19 (−1.1–1.47)	−0.35 (−1.4–0.69)	0.4 (−0.84–1.65)	−0.03 (−1.09–1.03)	0.31 (−0.94–1.55)
T3 MEHP	−0.1 (−1.18–0.97)		−0.46 (−1.52–0.6)		−0.41 (−1.48–0.67)	
Average MEHP	−0.2 (−1.48–1.08)		−0.43 (−1.69–0.83)		−0.25 (−1.52–1.03)	
T1 MEP	0.32 (−0.45–1.08)	**0.99 (0.13–1.86)**	0.18 (−0.58–0.94)	**1.01 (0.15–1.86)**	0.24 (−0.51–1)	**0.96 (0.11–1.8)**
T3 MEP	0.18 (−0.49–0.86)		0.31 (−0.36–0.99)		0.38 (−0.29–1.05)	
Average MEP	0.23 (−0.55–1.01)		0.25 (−0.52–1.02)		0.38 (−0.39–1.15)	
T1 MCPP	0.4 (−0.41–1.21)	0.47 (−0.45–1.38)	0.49 (−0.3–1.28)	0.48 (−0.41–1.36)	0.65 (−0.14–1.44)	0.64 (−0.24–1.52)
T3 MCPP	0.5 (−0.26–1.27)		0.41 (−0.35–1.16)		0.37 (−0.39–1.13)	
Average MCPP	0.59 (−0.3–1.47)		0.57 (−0.29–1.44)		0.61 (−0.26–1.47)	
T1 MiBP	0.61 (−0.62–1.85)	0.78 (−0.67–2.23)	0.61 (−0.62–1.83)	1.1 (−0.32–2.53)	0.88 (−0.36–2.12)	1.2 (−0.24–2.63)
T3 MiBP	0.26 (−0.84–1.36)		0.5 (−0.59–1.58)		0.66 (−0.43–1.74)	
Average MiBP	0.84 (−0.56–2.24)		1.09 (−0.3–2.47)		**1.44 (0.05–2.84)**	
T1 ∑DEHP	0.62 (−0.54–1.79)	1.35 (−0.05–2.74)	0.51 (−0.63–1.66)	**1.49 (0.13–2.84)**	0.73 (−0.43–1.88)	**1.52 (0.17–2.86)**
T3 ∑DEHP	0.99 (−0.26–2.24)		0.46 (−0.79–1.71)		0.2 (−1.06–1.46)	
Average ∑DEHP	1.23 (−0.15–2.62)		0.71 (−0.66–2.09)		0.59 (−0.79–1.97)	
T3 ∑DEHTP	0.66 (−0.23–1.56)		0.53 (−0.35–1.41)		0.61 (−0.26–1.48)	

Note: Models were adjusted for study center, race, age at delivery, household income, highest level of education, marital status, cigarette smoking in the first trimester, pre-pregnancy BMI, parity, gestational age, and antihypertensive use (where applicable). Significant results are shown in bold.
